# Predictive keywords: Using machine learning to explain document characteristics

**DOI:** 10.3389/frai.2022.975729

**Published:** 2023-01-05

**Authors:** Aki-Juhani Kyröläinen, Veronika Laippala

**Affiliations:** School of Languages and Translation Studies, University of Turku, Turku, Finland

**Keywords:** keyness, keyword, corpus linguistics, support vector machines, machine learning

## Abstract

When exploring the characteristics of a discourse domain associated with texts, keyword analysis is widely used in corpus linguistics. However, one of the challenges facing this method is the evaluation of the quality of the keywords. Here, we propose casting keyword analysis as a prediction problem with the goal of discriminating the texts associated with the target corpus from the reference corpus. We demonstrate that, when using linear support vector machines, this approach can be used not only to quantify the discrimination between the two corpora, but also extract keywords. To evaluate the keywords, we develop a systematic and rigorous approach anchored to the concepts of usefulness and relevance used in machine learning. The extracted keywords are compared with the recently proposed text dispersion keyness measure. We demonstrate that that our approach extracts keywords that are highly useful and linguistically relevant, capturing the characteristics of their discourse domain.

## 1. Introduction

Intuitively, some elements of a text are more important than others in informing readers about the text's characteristics. In corpus linguistics, this intuitive concept has been developed into a method that is referred to as keyword analysis (for recent overviews see Gabrielatos and Marchi, 2011; Egbert and Biber, 2019; Gries, 2021). Over the years, keyword analysis has become an instrumental part of quantitative text analysis in corpus linguistics as a way to examine the characteristics of various text varieties ranging from news articles to erotic narratives, through the contribution of words or other linguistic elements (see Gabrielatos and Marchi, 2011; Egbert and Biber, 2019, for a comprehensive overview of studies).

Recently, there has been an interest in methodological development of keyword analysis, as exemplified by such studies as Egbert and Biber (2019) and Gries (2021). The present study is situated against this backdrop. We present a new approach for a keyword analysis that is based on prediction rather than statistical calculation. We exemplify this approach by examining the characteristics of a corpus featuring two text varieties: news and blogs. By using linear support vector machines as classifiers, this approach allows us not only to predict the text variety associated with a given text, but also build inferences based on the learned mapping between the text variety and its linguistic characteristics.

Despite the long tradition of keyword analysis in corpus linguistics, it is surprising that there has not been many attempts to establish a systematic approach for evaluating the extracted keywords of a particular method. It is a common practice in quantitative studies to provide some measurement of goodness-of-fit. Recently, this sentiment was echoed by Egbert and Biber (2019) when they state: “While we believe these simple methods are useful for evaluating the various keyword methods, future research could explore more sophisticated metrics for evaluating and comparing keyword lists.” Similar situation can be found in NLP-based studies as well (for recent discussion, see Rönnqvist et al., 2022). At the same time, a large number of studies have examined different ways of taking into account the uncertainty of extracting keywords from corpora but not the quality of the extraction process itself. An excellent summary of various statistics used in keyword analysis is presented in Pojanapunya and Todd (2018). To evaluate the quality of the keywords, we develop rigorous, formal metrics to evaluate them, based on the well-established distinction between usefulness and relevance of variables applied in machine learning (see Guyon and Elisseeff, 2003) while maintaining a dialogue with the evaluations discussed by Egbert and Biber (2019).

Before discussing our proposed approach in detail, we briefly outline the central concepts of keyword analysis and how it has been operationalized previously in corpus linguistics. This provides us with the opportunity to better situate the proposed approach.

### 1.1. Keywords and keyness in corpus linguistics

Keyword analysis provides a means for a quantitative linguistic analysis of textual content. Mike Scott proposed a simple but effective definition that still provides the essential building blocks of keyword analysis: a keyword is a word that occurs with an “unusual frequency” in a target corpus compared with a reference corpus (Scott, 1997: 236, for discussion about various definitions of a keyword, see Stubbs, 2010). Hence, when defined in this manner, keyword analysis aims at identifying the words that are the most informative about the characteristics of a collection of texts relative to some other collection. In keyword analysis, the former collection of texts is referred to as the target corpus and the latter as the reference corpus.

The concept of text characteristics plays a critical role in keyword analysis. It can be understood broadly, covering various kinds of differences in the style, discourse domains, or functional characteristics that are expressed in the target corpus, or it can be interpreted in a more narrow sense, focusing on the “aboutness” of the target corpus, that is, on its main concepts, topics, or attitudes (cf. Williams, 1976; Phillips, 1989; Scott and Tribble, 2006; Bondi and Scott, 2010; Gabrielatos and Marchi, 2011).

More recently, Egbert and Biber (2019) argued that the focus of keyword analysis should be on aboutness, which is expressed in particular by content words–nouns and verbs that are relevant for the topics expressed in the texts. However, studies on text classification have demonstrated that focusing solely on topical words tends to lack generalizability to new texts because topics can vary substantially even within text categories such as news or encyclopedia articles (see Petrenz and Webber, 2011; Laippala et al., 2021). This is supported by the findings by Laippala et al. (2021), who showed that the inclusion of grammatical information can improve the generalizability of a model in text classification. Thus, focusing solely on aboutness may limit the generalizability of keyword analysis to the texts that just happened to be a part of the target corpus and share similar topics. On the other hand, if the analysis is primarily based on grammatical and function words, keyword analysis is unlikely to capture all the relevant characteristics of the texts because content words are also required to fully describe them. Thus, in our view, keyword analysis requires a careful consideration of both aboutness and other text characteristics to provide a full perspective to the important aspects of the texts–a point we will make throughout the current study and discuss in detail in the general discussion section.

In addition to the distinction between aboutness and other text characteristics, another aspect of keyword analysis that has gained a lot of attention recently is how to measure keyness, that is, how to extract the keywords from the bulk of words in the target corpus and determine the relative ranking of the keywords. There are two important aspects related to a traditional keyword analysis. First, traditional keyword analysis has relied on simple statistics, for example, a chi-squared test (Aarts, 1971), log-likelihood ratio (Rayson and Garside, 2000), and frequency differences (Gabrielatos and Marchi, 2011), among others. Second, traditional keyword analysis relies on frequency. However, there are a number of different ways in which the frequency of a word can be calculated. Traditionally, frequency is calculated based on the occurrence of a given word in the target and reference corpus. However, as Egbert and Biber (2019) have pointed out, calculating frequency in this manner does not take into account the individual texts used to compile the target and reference corpus (see also Gries, 2008). Hence, these methods analyze the potential differences only at the level of the target and reference corpus, without making any reference to the texts that may display a wide range of variation. To this end, Egbert and Biber (2019) proposed to determine keyness based on dispersion, that is, the number of documents a given word occurs in, and to use these dispersion measures of the target and reference corpora for a log likelihood estimation (for discussion about dispersion see also Gries, 2021). They referred to this measure as text dispersion keyness (TDK). The analysis presented in Egbert and Biber (2019) demonstrated that TDK could extract keywords of a high quality. Thus, we make use of this method as a point of comparison for the prediction-based approach proposed in the current study.

Although TDK takes into account the individual texts comprising the target and reference corpus, not all texts in a given corpus are equally good examples of their intended category. Instead, the situational and linguistic characteristics of the texts may vary so that, for example, not all news articles serve as the best possible exemplar of the news category. This observation has not just emerged from studies on text classification, where the classification performance can reflect this variation, but it can also be observed in inter-rater agreements in text annotation tasks (e.g., Egbert et al., 2015). To the best of our knowledge, none of the currently used methods in keyword analysis incorporate uncertainty as part of the extraction process or the computation of the keyness score. In the approach we propose, because of its predictive nature, we can take into account this variation, thus potentially improving the quality of the keywords. Importantly, although keyword analysis is widely used in corpus linguistics, there is no general approach in the current literature for evaluating the quality of the extracted keywords (see Egbert and Biber, 2019, for a recent discussion about the issue). It is possible to devise numerous different methods for evaluating the extracted keywords. The crux of the matter is, however, in grounding the methods used in the evaluation. In this manner, the concept of the quality of a keyword can also be precisely defined. Only through quantifying this concept can we begin to gain a better understanding of the preciseness of the keywords in describing the characteristics of a particular text. We pursue these topics in the following section, where we present the proposed approach for keyword analysis.

### 1.2. Present study

In the current study, we propose that keyword analysis could be considered a prediction problem (for general discussion about prediction see Breiman, 2001b; Shmueli, 2010, among others) rather than counting the frequency of the words in the texts and then performing a statistical test to evaluate the “unusually frequent” words. Thus, the goal of this approach is moved from comparing the frequency counts between the target and reference corpus to classifying the individual texts into a target corpus and reference corpus. This approach allows us to define keywords as those words that contribute to the discrimination between the two text classes, that is, the target corpus and reference corpus. Consequently, the concept of keyness also emerges naturally from this as the discriminative strength of a given keyword. Importantly, in this approach, the target corpus and reference corpus are not treated as homogeneous collections of texts, but each individual text is classified separately.

As we mentioned above, the current practice of keyword analysis lacks a general approach for evaluating the keyword quality, which is also discussed by Egbert and Biber (2019). Understanding the method as a prediction problem allows us to approach the evaluation with measures and concepts typically applied in machine learning. These are the concepts of usefulness and relevance (Guyon and Elisseeff, 2003), which hold a long tradition of evaluating variable selection in machine learning (e.g., Blum and Langley, 1997; Kohavi and John, 1997). In general, useful variables refer to the subset of variables that retain high predictive power, whereas relevant variables refer to the subset of variables providing a high descriptive adequacy of the categories under investigation.

In the present study, we combine usefulness and relevance into the concepts suggested by Egbert and Biber (2019) to evaluate keyness specifically. Thus, we examine usefulness of the estimated keywords through four concepts: 1) discriminability, 2) stability, 3) distinctiveness, and 4) generalizability. We present these concepts below and discuss relevance and its relation to keyword analysis.

First, the discriminative performance of the predictive model–to what extent the model discriminates between texts in the target and the reference corpora–gives a direct method for quantifying the usefulness of the keywords. The traditional count-based methods for estimating keyness do not allow for this kind of evaluation. From a purely technical perspective, there can be a number of standard metrics to carry out such an evaluation in machine learning. These are discussed in Section 5.2.

Second, stability refers to the consistency of the keywords toward minor changes in the target and reference corpus. This is crucial because the estimated keywords are a by-product of the corpus compilation process, which can result in deviations and biases that can affect the keywords (for discussion see Pojanapunya and Todd, 2018; Geluso and Hirch, 2019, and citations therein). We argue that useful keywords should also be stable in the face of subtle changes to the makeup of the corpora (e.g., Laippala et al., 2021). We demonstrate that in a prediction-based approach for keyness, incorporating a measure of stability is straightforward and simple, see Section 5.2 for details.

A third aspect of usefulness examined in the current study is distinctiveness. This refers to the extent to which the keywords reflect the characteristics of their target corpus as opposed to the reference corpus. Furthermore, keywords do not simply represent an unstructured list of words; their ordering is expected to mirror their relation to the target corpus. Interestingly, Egbert and Biber (2019) advocated for this type of relation, which they coined as content distinctiveness. They defined it in the following manner:

Content-distinctiveness refers to the strength of the relationship between a keyword and the content of the discourse domain represented by the target corpus […]. (Egbert and Biber, 2019: 236)

The fourth aspect related to the usefulness of the extracted keywords is generalizability. The primary focus of keyword analysis is to provide insights not only into the characteristics of the specific texts in the target corpus, but also into new texts representing the same discourse domain as the target corpus. For a prediction-based approach, generalizability is easily quantified by evaluating the discriminative performance of the model on new texts, but such an evaluation cannot be used with traditional keyword analyzes. To compare the generalizability of the estimated keywords between a traditional and prediction-based method, we propose a new metric–lexical coverage–to reflect the proportion of keywords attested in new texts representing the same discourse domain as the target corpus.

Finally, usefulness is an important aspect in understanding and evaluating the quality of the keywords, but at the same time, it is only one side of the coin–the other side is relevance. In keyword analysis, relevance refers to the degree to which the keywords are representative and meaningful in relation to their corresponding target corpus and the discourse domain it represents. Similar argumentation can be found in Egbert and Biber (2019), in which they emphasize the importance of relevance in evaluating the quality of the keywords, specifically the importance of content words over function ones.

Importantly, in machine learning research, usefulness and relevance can be seen as competing strategies in optimizing the informativeness of a given method (e.g., Blum and Langley, 1997; Kohavi and John, 1997; Guyon and Elisseeff, 2003). In principle, a method can be extremely useful, that is, display a high discriminative performance while simultaneously demonstrating low relevance. For example, Ribeiro et al. (2016) showed that usefulness alone cannot be used to judge the merits of a method. They trained two classifiers to discriminate two text categories —“Christianity” and “Atheism”—from the widely used 20 newsgroup dataset (http://qwone.com/~jason/20Newsgroups/), with one based on the original unprocessed corpus and another one on a preprocessed version. In the preprocessed version, elements low on relevance were removed. These included the author names and header information of the web pages, among other things.

The performance of the classifier trained on the preprocessed corpus was 88.6% compared with 94.0% achieved by the model trained on the unprocessed corpus. Based on the discriminative performance alone, that is, usefulness, the model trained on the unprocessed data would be chosen as the “best.” However, the better-performing classifier was based on features that were not relevant to the categories of “Christianity” and “Atheism.” This was confirmed when the classifiers were evaluated against a new dataset that consisted of similar newsgroup texts but from different sites. In this setting, the discriminative performance was reversed–the preprocessed model achieved an accuracy of 69.0%, while the model trained on the original unprocessed data had an accuracy of 57.3%. This clearly shows the motivation behind our evaluation approach. The discriminative performance of a model alone cannot be the metric to evaluate its goodness: generalizability and relevance must be taken into consideration.

Thus, far we have charted a general approach for evaluating the quality of keywords. To implement a prediction-based approach to keyness, a machine learning algorithm, however, is required. The proposed approach presented in the current study is flexible and not restricted to a specific machine learning algorithm. There are hundreds of algorithms to choose from for a classification task alone (Fernández-Delgado et al., 2014). Even in linguistically-oriented studies, there are a number of classical machine learning algorithms such as random forests which tend to perform extremely well modeling tabular data (Fernández-Delgado et al., 2014). They have also been used to model linguistic data such as dialectal variation (Tagliamonte and Baayen, 2012), eye-movements during reading (Matsuki et al., 2016) and phonological variation (Arnhold and Kyröläinen, 2017). In NLP, deep-learning neural networks and specifically transformer-based architecture has effectively become the standard approach for modeling linguistic data (Devlin et al., 2018; Conneau et al., 2020).

A keyword analysis when framed around machine learning, however, does not rest on discriminative performance alone but, by necessity, requires that the decisions of the implemented architecture can be examined. While contemporary machine learning algorithms can provide excellent discriminative performance, one of the challenges facing their utilization is to understand which of the variables and how they affected the discriminative performance (Samek et al., 2017). Indeed, a large number of different methods have been proposed in order to explain the decisions of a given model (Montavon et al., 2018; Linardatos et al., 2020). However, these methods tend to focus on explaining individual data points not categories such as registers (for a recent overview see Rönnqvist et al., 2022).

Given this background, we implemented the proposed approach using linear support vector machines (SVMs) (Vapnik and Vapnik, 1998). SVMs are widely used and have demonstrated excellent performance, ranging from classification to regression problems in a number of different scientific fields (Schölkopf et al., 2002). They also have a long tradition in text classification because this task tends to present difficulties for machine learning algorithms due to the extremely high dimensionality of the data—see Section 5.1—but SVMs can learn independent of the dimensionality of the data (see Joachims, 1998). Although SVMs in general are primarily used for prediction, linear SVMs can also be used for the purposes of inference (see Guyon et al., 2002; Zhang et al., 2006; Richardson and Campbell, 2007; Sharoff et al., 2010; Wang et al., 2019). Laippala et al. (2021) uses linear SVMs to explore the importance of lexis and grammar to model text varieties in English.

Here, we continue this line of investigation where the analysis utilizes linear SVMs. From a methodological point of view, we demonstrate that estimations obtained with linear SVMs can be directly linked to a specific text variety providing precise inference without post-processing. The data used in the current study are described in Section 2. Given the simpler model architecture of linear SVMs (see Section 3), it is possible that the proposed method might be associated with lower discriminative performance compared to other machine learning algorithms. In order to better situate the implemented method, we also modeled the data using random forests and a deep language model, BERT, see Section 4. In Section 5, the steps for preprocessing the data and model fitting are explained in detail. To ground the results relative to traditional keyword analysis, we analyzed the data used in the study with TDK. The evaluation of the keywords in terms of their usefulness and relevance is presented in Section 7.

## 2. Data

The data used in the current study were extracted from the Corpus of Online Registers of English (CORE) Biber and Egbert (2015). CORE is currently the largest collection of English online texts (*N* = 48,571) with manually annotated information pertaining to text variety. The texts in CORE were collected based on a large number of pseudo-random Google searches, with the aim of capturing a representative sample of the variation of online language use. Importantly, CORE is not limited to a set of predefined text varieties but instead attempts to cover the full range of linguistic variation found online. The annotation scheme is a hierarchical taxonomy created in a data-driven manner, consisting of eight general categories and 33 subcategories. Each text was classified using four annotators, with a majority vote used to determine the final category of a specific text. A detailed discussion and description of the annotation process and the taxonomy are provided in Biber and Egbert (2015) and Biber and Egbert (2018), respectively.

In general, text varieties are associated with a specific situational context and give rise to important differences in language use (Biber, 2012). For the purposes of the present study, we focused on two varieties: news articles and personal blogs, or news and blogs for short. The use of these two text varieties has a number of benefits. First, this allowed us to directly compare the results with traditional keyword analysis, namely the TDK proposed by Egbert and Biber (2019) and discussed in Section 6. Second, previous studies have shown that these two text varieties are well defined in terms of their situational and linguistic characteristics. This ensures that evaluating the relevance of the keywords becomes easier because they can be anchored relative to previous studies (e.g., Biber and Egbert, 2016, 2018). In turn, this allows for a more reliable evaluation of the proposed method (Biber and Egbert, 2018; Laippala et al., 2021). For the purposes of the present study, we compiled two corpora based on CORE. The primary corpus was larger and was used in training the linear SVMs and calculating the TDK. Furthermore, this dataset allowed us to evaluate the usefulness and relevance of the extracted keywords. The secondary corpus was specifically formed to test the generalizability of the extracted keywords to new texts. We separately describe the composition of these corpora below.

In the case of the primary corpus, we randomly sampled 1,000 texts for each variety. Based on our prior experience in text classification, the size of the data was large enough to provide stable estimates (e.g., Laippala et al., 2021). However, whereas in traditional keyword analysis the reference corpus typically consists of a significantly larger collection of texts than the target corpus, we balanced the number of texts between the two varieties. We did this because in a classification task, a substantial imbalance between the classified categories impacts the model performance, and we were aiming to ensure that a possible difference in the classification performance between the text varieties was not attributable to the size of the respective corpus. This setting is sometimes referred to as a cross-corpus comparison in corpus linguistics. The summary information of the dataset used in the present study is provided in Table 1.

**Table 1:** Summary information of the primary and the secondary corpus.

	**Number of texts**	**Number of words**	**Number of (word) types**
**Primary corpus**			
Blogs	1,000	1,237,574	41,938
News	1,000	982,271	39,828
**Secondary corpus**			
Blogs	100	138,258	11,332
News	100	105,093	11,315

As outlined in Section 1.2, we extracted another random sample of texts from CORE in order to examine the generalisability of the keywords. This secondary corpus was used only for prediction in Section 7.3. This second sample consisted of a total of 200 new texts, split evenly between news (*n* = 100) and blogs (*n* = 100). The summary information of this secondary corpus is given in Table 1.

## 3. Support vector machines

In this section, we outline the conceptual basis of SVMs when they are used in a binary classification and, specifically, how they can contribute to keyword analysis. The learning mechanism of SVMs is based on the fundamental idea of finding the optimal boundary that separates two categories by a maximal distance. This is referred to as an optimal hyperplane (line in 2D, plane in 3D and hyperplane in more than three dimensions). However, there are potentially several different hyperplanes that could be used to separate the two categories in a given dataset. To find the optimal one, SVMs use observations from both of the categories closest to the hyperplane. These observations along with their features are called support vectors because they support the hyperplane and are considered to be representative exemplars of their corresponding category (Vapnik and Vapnik, 1998; Schölkopf et al., 2002).

**Figure 1:**
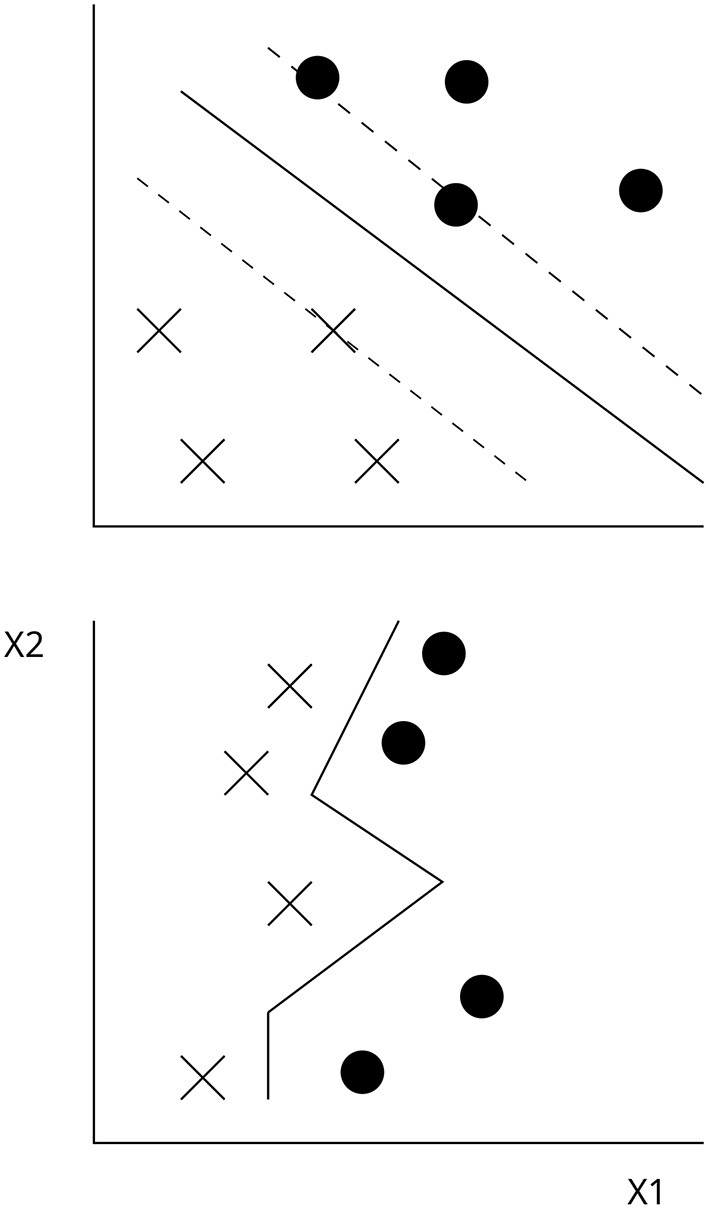
Illustration of SVMs and the linear separation of the categories, dots and crosses, **(upper)** and generalizability **(lower)** in a binary classification.

The goal of the algorithm is to maximize the distance separating the two categories, which are referred to as the margin and optimal hyperplane, for a given data. This conceptual basis of SVMs is illustrated in Figure 1 (upper), in which the binary response variable consisting of dots and crosses is modeled as a function of two predictors (X1 and X2). The solid line represents the hyperplane, and the dashed lines correspond to the maximal margin.

In text classification, a given text is represented as a vector consisting of feature-response pairs where each word corresponds to a feature, the value of a given feature is its frequency in a given text, and the text variety of a given text is the response. See Section 5 for more details. To learn the mapping between the features and the response, SVMs are trained on these feature-response pairs. Formally, SVMs require the solution to the following optimization problem (Boser and Guyon, 1992; Cortes and Vapnik, 1995):


$$\begin{array}{l} \frac{min}{w,b,\xi }\frac{1}{2}w^{T}+C\overset{l}{\underset{i=1}{\sum }}\xi_{i} \end{array}$$

$$\begin{array}{l} subject\;to\;y_{i}[w^{T}\phi (x_{i})+b]\geq 1-\xi_{i},\\ \;\xi_{i}\geq 0 \end{array}$$

The features are given as *x*_*i*_ and the response variable is *y*_*i*_∈{−1, +1}. In the case of a linear SVMs, the decision function is a combination of *w*, *b*, and ξ_*i*_. We go over the terms used in the decision function separately below because they have important consequences for building precise inferences with linear SVMs.

A special term in SVMs is the bias (*b*), which ensures that the separating hyperplane is estimated with the maximal margin by shifting the decision function down or up. Hence, the bias is scalar and is calculated as the average over the weights that satisfy the strict inequality, here for a given weight α, 0 <α <*C* (Guyon et al., 2002). The third term used in the decision function is ξ_*i*_, that is, a slack variable ensuring convergence of the algorithm in case of nonlinearly separable data (Schölkopf et al., 2002).

In the formulation, the term *C* is a hyperparameter controlling the trade-off between the classification accuracy and generalizability of the model. A model that follows the data too closely may have a high accuracy but may not generalize well to new data. Conversely, a less flexible model may have a lower accuracy on the training data but can achieve a higher accuracy on new data. This idea is illustrated in Figure 1 (lower) where the wigglyness of the decision boundary is affected by C. Importantly, because this is a hyperparameter, the model cannot learn it from the data. Hence, it must be tuned by the modeler, and its value depends on the data at hand. See Section 5 for a discussion on this.

In terms of inference and the proposed keyword analysis, the weight vector *w* is of primary interest. In the case of this study, a given feature of the weight vector corresponds to a word used in the modeling. Most of the weights in the vector—the frequencies of the feature in a particular text—are zero, and only a nonzero weight can affect the decision function in SVMs (Guyon et al., 2002). Importantly, the direction of the weights, whether positive or negative, indicates on which side of the hyperplane a given observation is going to be located, hence determining which of the two categories is going to be the model prediction for that particular instance. This allows us to associate a given feature with a particular text variety in the current study.

The final important aspect of SVMs for the purposes of the present study concerns the mapping learned by SVMs. This mapping is learned based on the observations used in the training of the model (*x*_*i*_) and the function ϕ. This function is referred to as a kernel function $K\left(x_{i},x_{j}\right)\equiv \phi {\left(x_{i}\right)}^{t}\phi \left(x_{j}\right)$. In the case of a linear function, this kernel is given as $K\left(x_{i},x_{j}\right)=x_{i}^{T}x_{j}$.

In sum, we have presented a general overview of linear SVMs and how this algorithm learns a mapping between the features and response variable. We argue that this conceptual basis of linear SVMs can lead to keyword analysis where a given weight corresponds to the keyword and its magnitude to keyness. Before empirically testing this postulation, we turn to the practical side of modeling the data with SVMs. Finally, because we are only using linear SVMs, we henceforth refer to them simply as SVMs.

## 4. Random forest and deep language model

In order to situate the discriminative performance of linear SVMs, we also modeled the data using random forests and the deep language model BERT. As the main focus of this study is in the methodological approach of evaluating keywords, we only briefly describe these two algorithms in this section.

Random forests were developed by Breiman (2001a) and are based on a large collection of classification and regression trees (CART). They are a non-parametric method, making them highly suitable for modeling non-linear data contrasting linear SVMs. This is also often the case with linguistic data. While CART recursively partitions the data based on binary splits into increasingly more homogenous categories, random forests introduce randomness to this process. First, a given tree is trained on a random sample of the data. Second, only a pre-predefined number of potential predictors is randomly selected at a given split used to partition the data. This is the primary hyperparameter of the model. In comparison to other classical machine learning algorithms, random forests are widely used in different areas of scientific research and tend to produce excellent results with minimal fine-tuning of the model (see Fernández-Delgado et al., 2014).

BERT, Bidirectional Encoder Representations from Transformers (Devlin et al., 2018) is a language model following the transformer architecture (Vaswani et al., 2017) and trained on large amounts of data from Wikipedia and books. The model can be fine-tuned to downstream NLP tasks, such as text classification, and it has been shown to achieve important improvements and state-of-the-art results for instance in register identification (Repo et al., 2021). This performance advantage does, however, come with an increase in computation time and model complexity.

Finally, it is important to mention that, unlike BERT, random forests provide a built-in mechanism for estimating relative variable importance. However, this is a global measure and it does not provide information about the direction of the effect. As a drawback, both random forests and BERT would require implementing some post-processing techniques in order to derive a measure of variable importance that was sensitive to a specific category. This is an especially complex problem for deep neural networks (for discussion see Rönnqvist et al., 2022). For this reason, we trained these models only to situate the discriminative performance of the linear SVMs.

## 5. Methodological solutions

In this section, we discuss the methodological solutions we have taken during data preprocessing and model fitting. The chosen representation of the data is discussed first because it is concerned with the fundamental basis of the analysis. The second part of this section covers the model-fitting process of the implemented SVMs. Throughout the current study, Scikit learn (version 0.21.1) was used along with Python3. The statistical analyzes and data visualization were carried out in R, version 4.1.1 (R Core Team, 2021).

### 5.1. Data preprocessing

In principle, keyword analysis can be based on any unit attested in a text. Indeed, in previous studies, a number of different units have been examined ranging from words, lemmata, *n*-grams and part-of-speech information to larger lexicogrammatical patterns (see Gabrielatos, 2018 for discussions and citations therein).

In the present study, we focused solely on the contribution of words, specifically a bag-of-words (BOW) representation, where each distinct word was considered a feature. This decision was taken for two reasons. First, Laippala et al. (2021) compared seven different feature sets ranging from words and grammatical features to character *n*-grams, showing that although the combination of grammatical and lexical information provided the best discriminative performance between text varieties, word-level information alone was highly competitive. Second, traditional keyword analysis is primarily concerned with word-level BOW information. This makes it easier to compare the results to previous keyword studies on text characteristics. To exclude linguistically unmeaningful features and reduce the dimensionality of the BOW representation, we deleted numbers and punctuation and normalized letters to lower case. The resulting BOW consisted of 1,935,316 words (54,693 types) and it was used to train the SVMs.

Although the values of the word-level features typically correspond to frequency, that is, the number of times a given word appeared in a particular text or corpus, it is nonetheless open to different quantifications. Because Egbert and Biber (2019) have recently brought forth the advantages associated with quantifying frequency in different ways (see Gries, 2008 for a comprehensive summary), we also considered adjusting the absolute word frequencies but in our case using term frequency-inverse document frequency (tf-idf) weighting. Tf-idf is widely used in natural language processing and information retrieval (Spärck, 1972). Here, the value is increased when a word occurs frequently in a small number of texts and decreased when a word occurs in a large number of texts. Thus, this adjusted frequency gives more importance to words that potentially discriminate among texts in the dataset. To compare the usefulness of the frequency weighting, we created two distinct versions of the data using the vectorizers available in Scikit learn: CountVectorizer to obtain absolute word frequencies and TfidfVectorizer to obtain tf-idf-weighted word frequencies. Finally, both BOWs were L2 normalized.

The final preprocessing step was implemented to examine the distribution of content and function words among the extracted keywords. Recently, this issue has been raised by Egbert and Biber (2019) in their comparative study on traditional keyword methods (see Section 1). This allows us to examine the degree to which a particular keyword method is likely to display sensitivity toward differences in text characteristics rather than in aboutness. To examine the proportion of content and function words among the keywords, we parsed the data using Turku Neural Parser (Kanerva et al., 2018), here following the Universal Dependency Schema (Nivre et al., 2016). The parsed output was used to determine the part-of-speech (POS) classes of the keywords used in Section 7.4.

The POS associated with a particular word can vary depending on the context in English. For this reason, the analysis was based on the dominant POS (most frequent tag) associated with a given word, which is similar to Brysbaert et al. (2012). From the POS information, we formed the lexical class function word consisting of adpositions, conjunctions, pronouns, and auxiliaries. Finally, nouns, verbs, and adjectives were kept as lexical classes of their own, and the remaining POS were merged into a category labeled other.

### 5.2. Model fitting and evaluation

For the purposes of modeling the data with SVMs to extract keywords, the preprocessed BOW data were split into training (80%) and test (20%) sets. In addition to producing an extremely high dimensional space, BOW also generates a representation that is extremely sparse because most words do not occur in every text. Hence, they have a frequency of zero in those instances. To reduce this sparsity, all words with a dispersion of <5% of the training data were removed. Although this is an arbitrary choice and the application of a cut-off point is known to affect keywords (Egbert and Biber, 2019; Pojanapunya and Watson Todd, 2021), we demonstrate in Section 7.1 that even the current cut-off point of 5% generated keywords that can be regarded as highly unstable.

The model fitting procedure was implemented with the SVC package with a linear kernel. Prior to training the SVMs, the hyperparameter *C* of the linear kernel had to be tuned because it significantly impacts performance. For both the absolute and weighted frequency data, the optimal value of *C* was 0.1 for both models, and it was found *via* grid search within the range of 0.001 and 10. The same value of *C* was used across the resampling.

The model fitting procedure was then implemented in the following way: (a) The SVMs were trained on the training data separately for the absolute and weighted frequency BOW using the optimal value of *C*. (b) The discriminative performance of the SVMs were evaluated on the test data with three measures: precision, recall, and F1-score (the harmonic mean of precision and recall). (c) The top 1,000 positive and negative weights were extracted from the model. (d) The data were randomly resampled into training (80%) and test (20%) sets. The model fitting procedure started from the beginning, and this procedure was repeated 1,000 times.

Resampling allowed us to directly quantify the (in)stability of the model and of the extracted keywords toward small changes in the data. Additionally, Laippala et al. (2021) have shown that the extraction of 1,000 weights is sufficient in practice as this procedure already yields a large number of keywords that tend to be unstable (see also Section 7.1 for similar results). We will refer to this measure as selection frequency. Although a keyword analysis is only typically based on a subset of the top ranking keywords that are often limited to the top 100 keywords (see Pojanapunya and Todd, 2018, for a comprehensive analysis of previous studies), the use of 1,000 positive and negative weights provides a larger number of keywords. This allowed us to evaluate the stability of the keywords. A lower selection frequency implies that the keywords depended on just some part of the data, such as topical or idiosyncratic properties of the data. A higher selection frequency, on the other hand, suggested that the keywords represented stable characteristics of their corresponding text variety and could be generalized to the entire corpus.

The following procedure is implemented for tuning the hyperparameters with random forests and BERT. For BERT, we used use the large version of BERT with a batch size of 8 in the Huggingface library (PyTorch version) and ran a grid search from 0.00001 to 0.01 to optimize the learning rate. For the random forest, we used the RandomForestClassifier in Scikit learn Pedregosa et al. (2011), optimizing for the number of trees with a grid between 500 and 2,000.

## 6. Text dispersion keyness

To evaluate the keywords extracted with SVMs, we used the recently introduced TDK as a point of comparison. This method is described in Egbert and Biber (2019) and also recently discussed in Gries (2021). This measure is based on text dispersion by comparing type frequencies–in how many texts a word occurs in the target and reference corpora. Although the TDK is based on the observed type frequency (*O*), the keyness score is based on the log-likelihood ratio (LLR) (see Dunning, 1993). Accordingly, the expected frequency is calculated in the following manner:


(1) $$\begin{array}{l} E_{i}=\frac{N_{i}\sum_{i}O_{i}}{\sum_{i}O_{i}} \end{array}$$

The LLR is calculated as follows:


(2) $$\begin{array}{l} -2ln\lambda =2\underset{i}{\sum }O_{i}ln\left(\frac{O_{i}}{E_{i}}\right) \end{array}$$

Because this method represents the traditional approach to keyword analysis where keyness is calculated for a target corpus relative to a reference corpus, we calculated the LLR score for both news and blogs separately, changing their roles as a reference and target corpus. Doing this made it possible to directly compare the results of the TDK with SVMs. At the same time, it should be noted that in a traditional keyword analysis, the reference corpus tends to be significantly larger than the target corpus (for a recent discussion about the influence of the reference corpus, see Pojanapunya and Watson Todd, 2021). For example, Biber and Egbert (2018) presented a keyword analysis in which the reference corpus consisted of all the text varieties attested to in CORE, except the one used as the target corpus. This makes their study design a little different from our binary setting and can also affect the estimated keywords. However, we wanted to keep the setup of extracting the keywords with the TDK as similar as possible to the SVMs to compare the results.

## 7. Results

A total of 4,524 keywords were estimated with the SVMs (tf-idf) through the implemented resampling procedure. Of these, 2,243 were associated with news and 2,281 with blogs, respectively. To illustrate the keywords and their estimated weights, the top 100 keywords for news are given in Table 2 and for blogs in Table 3. As can be seen, the keywords seem linguistically motivated.

**Table 2:** Top 100 keywords extracted with SVMs for news in descending order based on the estimated weights averaged across the 1,000 resamplings.

	**Weight**			**Weight**			**Weight**	
**Keyword**	***M***	***SD***	**Keyword**	***M***	***SD***	**Keyword**	***M***	***SD***
said	1.202	0.0331	ms	0.2172	0.0233	data	0.1544	0.0245
the	0.6628	0.0559	party	0.2164	0.0271	latest	0.154	0.0142
he	0.5885	0.0489	are	0.2122	0.0367	britain	0.1527	0.0161
his	0.5312	0.0413	romney	0.2112	0.0336	office	0.1524	0.0164
says	0.4751	0.03	against	0.2025	0.0171	awards	0.1518	0.0188
has	0.4662	0.0271	group	0.2022	0.0206	director	0.1488	0.0155
people	0.4407	0.0326	year	0.2002	0.0276	american	0.1468	0.0228
government	0.428	0.0227	added	0.1984	0.016	former	0.1466	0.0136
in	0.3651	0.0434	film	0.1953	0.0375	users	0.1457	0.016
s	0.3485	0.0434	british	0.1931	0.0211	factor	0.1448	0.0227
its	0.3433	0.0231	cameron	0.1867	0.0245	staff	0.1431	0.0178
mr	0.326	0.0348	hospital	0.1867	0.0249	smith	0.1425	0.0249
million	0.3202	0.0213	apple	0.1864	0.0329	she	0.1424	0.0482
who	0.3167	0.0269	be	0.1844	0.0321	celebrity	0.1423	0.0265
police	0.3117	0.0239	percent	0.1825	0.0184	judge	0.1416	0.0166
their	0.3016	0.0309	cookies	0.181	0.027	announced	0.1405	0.0128
by	0.2994	0.0279	movie	0.1801	0.029	should	0.1399	0.022
they	0.2973	0.0396	pay	0.1787	0.0196	city	0.1393	0.0295
of	0.2876	0.0486	business	0.1777	0.0254	network	0.1375	0.0245
will	0.2811	0.0327	report	0.1758	0.0186	officers	0.1367	0.0204
public	0.2756	0.0203	per	0.1743	0.015	policy	0.1357	0.0135
obama	0.2717	0.0321	money	0.1741	0.0269	financial	0.1336	0.0169
an	0.2511	0.0254	sex	0.1708	0.0225	reports	0.1336	0.0162
fire	0.2436	0.0281	evidence	0.1651	0.0166	nuclear	0.133	0.0305
news	0.2393	0.0257	admitted	0.1636	0.0167	is	0.1328	0.0408
president	0.2388	0.0223	loading	0.1634	0.0233	community	0.1324	0.0201
told	0.2368	0.0209	industry	0.1633	0.0143	media	0.1322	0.0238
company	0.2321	0.0194	national	0.1623	0.0165	states	0.1321	0.0139
star	0.2304	0.0212	economy	0.1608	0.0195	houston	0.1319	0.0302
minister	0.2273	0.0177	including	0.1601	0.0151	military	0.1305	0.0237
according	0.2244	0.0146	companies	0.1597	0.0171	revealed	0.1304	0.0141
state	0.2177	0.0179	women	0.1595	0.0284	would	0.1295	0.0291
fans	0.1581	0.0241	security	0.1561	0.0201			
cent	0.1576	0.0143	court	0.156	0.0221			

**Table 3:** Top 100 keywords extracted with SVMs for blogs in descending order based on the estimated weights.

	**Weight**			**Weight**			**Weight**	
**Keyword**	***M***	***SD***	**Keyword**	***M***	***SD***	**Keyword**	***M***	***SD***
i	–3.5114	0.0431	had	–0.2479	0.0286	much	–0.1866	0.0203
my	–2.1403	0.0415	one	–0.2477	0.0269	rain	–0.1855	0.0317
me	–1.079	0.0317	good	–0.2458	0.0267	like	–0.1851	0.0271
you	–1.018	0.049	too	–0.2421	0.0204	very	–0.1819	0.024
and	–0.9144	0.0474	about	–0.2408	0.0279	night	–0.1808	0.0285
so	–0.7613	0.0309	there	–0.2407	0.03	beautiful	–0.1806	0.019
we	–0.748	0.0572	back	–0.2395	0.0268	pretty	–0.1804	0.0186
it	–0.6958	0.0451	post	–0.2343	0.0231	nt	–0.1801	0.0339
was	–0.571	0.0459	always	–0.2335	0.0196	though	–0.179	0.0177
a	–0.5465	0.0523	room	–0.2316	0.0267	them	–0.1772	0.0294
blog	–0.5387	0.0286	days	–0.2314	0.0234	wonderful	–0.1758	0.0171
our	–0.5036	0.041	d	–0.2294	0.0263	lots	–0.1748	0.0183
am	–0.4302	0.0302	pages	–0.2236	0.0213	write	–0.1727	0.0173
day	–0.427	0.0289	bit	–0.2231	0.0244	went	–0.1717	0.0205
this	–0.4207	0.0358	life	–0.2204	0.028	boys	–0.1712	0.0244
some	–0.4086	0.0293	how	–0.2196	0.0233	ll	–0.1704	0.0237
all	–0.3847	0.0294	know	–0.2147	0.022	going	–0.17	0.0237
your	–0.3754	0.0343	what	–0.2102	0.0296	god	–0.1697	0.0244
up	–0.3749	0.026	work	–0.208	0.0295	week	–0.1696	0.028
love	–0.3507	0.0281	thanks	–0.2027	0.0188	happy	–0.1677	0.0174
lovely	–0.3335	0.0206	friend	–0.2024	0.0192	house	–0.1677	0.0288
things	–0.3288	0.0218	out	–0.1999	0.0277	sleep	–0.1664	0.0188
little	–0.3247	0.0278	fun	–0.1993	0.02	came	–0.1648	0.02
few	–0.3138	0.0232	but	–0.199	0.0284	quilt	–0.161	0.03
did	–0.3023	0.0255	got	–0.1986	0.0284	sure	–0.158	0.0182
time	–0.2948	0.0254	pink	–0.197	0.0297	reading	–0.1577	0.0205
to	–0.2922	0.0519	well	–0.1954	0.0215	way	–0.1575	0.0222
really	–0.2766	0.0245	book	–0.1949	0.0254	cake	–0.1569	0.0328
just	–0.2751	0.0256	go	–0.1946	0.0224	home	–0.1567	0.0275
great	–0.2739	0.0239	myself	–0.1946	0.0194	looking	–0.1548	0.0247
morning	–0.2643	0.0245	read	–0.1938	0.0298	remember	–0.1544	0.0203
here	–0.2613	0.0244	trip	–0.1904	0.0201	weeks	–0.1544	0.0206
then	–0.2563	0.0252	busy	–0.1873	0.0171			
get	–0.2522	0.0264	thank	–0.1872	0.0191			

For generating the keyword list with the TDK, previous studies have used different cut-off values on the LLR scale to trim the number of extracted keywords, for example an LLR score of 3.84 corresponds to a significance level of 0.05 or a score of 6.63 corresponding to a significance level of 0.01 (see Stubbs and Tribble, 2006). A different approach was taken in the current study because one of the goals of the analysis was to evaluate the stability of the estimated keywords. For this purpose, we required a larger number of keywords. Hence, a cut-of point of five was used. Typically, the results of a keyword analysis are based on the top 100 keywords. In this scenario, the application of a cut-off point does not affect the selection of the top ranking keywords but, naturally, will affect the total number of extracted keywords. Altogether, 2,134 keywords for blogs and 1,906 for news were extracted with TDK.

For the purposes of presenting the keywords, only the top 100 keywords, along with their keyness scores, are provided for blogs in Table 4 and for news in Table 5. The full list of the keywords are provided as separate files and are publicly available at https://osf.io/mxrt5/?view_only=3f4ceb05dc81413aaf1ff6c0d4b71aab.

**Table 4:** Top 100 keywords for blogs in descending order estimated with the TDK.

**Keyword**	**Keyness**	**Keyword**	**Keyness**	**Keyword**	**Keyness**
my	321.2696	felt	87.662	trip	67.3703
me	290.9727	life	86.6883	did	67.1608
blog	278.7961	remember	85.6759	thoughts	66.9537
love	217.5413	mom	83.3293	went	66.8936
myself	200.7759	your	82.7448	walk	66.3623
lovely	190.7492	mine	82.4932	baby	65.4903
am	168.2562	glad	80.7067	friends	65.4594
things	155.1241	thing	80.254	cute	65.1083
bit	140.4189	ll	79.9341	lunch	64.8648
feeling	134.7227	amazing	79.7949	here	64.7497
little	133.4441	bed	79.1399	thinking	64.7449
fun	131.9454	thanks	78.67	pages	64.5532
really	124.7018	busy	78.4548	photos	64.5473
write	117.0519	just	78.4447	kids	63.7531
feel	112.3408	few	78.3148	you	63.7188
awesome	109.8288	loved	78.077	read	63.491
sure	102.5493	thought	78.0398	writing	63.0943
oh	102.2059	try	75.005	going	62.0897
pretty	101.9557	lots	74.9351	getting	61.5805
too	101.7355	so	73.9009	ok	61.045
happy	100.1452	though	73.0248	dad	60.9457
nice	99.475	something	72.6951	like	60.3088
always	98.6983	beautiful	71.6701	wanted	60.2718
got	98.6623	great	71.6333	find	59.676
wonderful	95.5234	book	71.0861	seemed	59.587
friend	95.2765	good	71.0501	then	59.4047
know	93.6784	chocolate	70.9737	think	59.2791
maybe	93.5966	sleep	70.6114	dinner	58.8316
reading	92.454	quite	69.6334	excited	58.7534
day	91.7389	go	69.0343	hello	58.5443
stuff	90.769	much	69.0261	sweet	58.0425
thank	90.7014	hope	68.6257	sometimes	57.6348
post	88.9952	morning	67.4354		
hi	87.8674	posts	67.3731		

**Table 5:** Top 100 keywords for news in descending order estimated with the TDK.

**Keyword**	**Keyness**	**Keyword**	**Keyness**	**Keyword**	**Keyness**
government	239.7305	federal	67.5111	countries	49.7396
president	159.5535	sector	67.4284	members	49.6112
according	148.419	campaign	66.4956	regions	48.7573
said	122.997	election	66.092	services	48.3762
minister	121.9295	chairman	65.6403	citizens	47.2791
national	118.394	financial	65.4903	foreign	46.9301
global	112.164	leadership	64.7398	authorities	46.6795
million	110.818	against	64.5173	association	46.4406
chief	96.8648	director	64.4824	nation	46.2657
billion	96.3611	police	63.7239	development	45.8192
announced	96.2089	news	63.4357	investigation	45.6929
public	94.4555	court	63.1812	per	45.4764
reported	92.2906	companies	62.6363	based	45.3917
officials	91.974	british	62.5576	republican	45.0074
percent	91.4529	economy	61.5987	commission	44.5364
including	88.5112	washington	59.6809	among	44.5216
states	87.6227	prime	58.8316	administration	44.3335
reports	86.0951	industry	58.1103	country	43.6308
united	86.0258	leaders	57.9272	largest	43.5868
report	85.6594	senior	57.2007	leader	43.5377
economic	84.438	data	55.9019	rights	43.4038
committee	84.431	growth	55.4799	operations	43.3279
secretary	84.1851	says	55.0089	latest	43.045
policy	83.2111	governments	54.8837	mp	42.994
spokesman	81.7914	legal	54.1614	alleged	42.8423
its	81.5598	added	53.8647	proposed	42.7435
former	75.3566	agency	52.3396	source	42.5329
council	73.7053	revenue	51.4515	guardian	42.235
obama	73.2148	barack	51.2943	david	42.0362
political	72.1832	parliament	51.2943	officer	41.9696
state	71.831	britain	50.6955	kashmir	41.5888
security	71.5088	mr	50.56	dal	41.5888
international	70.4641	evidence	50.4145		
cent	68.9288	management	50.0988		

### 7.1. Usefulness: Discriminability and stability

Discriminability refers to how useful the data representations—the keywords—were in discriminating the classes, and stability relates to how stable the representations and, thus, the keywords were toward small changes in the data introduced by the 1,000 resampling rounds. As data representations, we compared the two BOW settings we introduced in Section 5.1, with one using absolute word frequency and the other using word frequency weighted with tf-idf. We evaluated which one of the two BOW representations provided a better fit to the data and, consequently, was shown to be more useful in discriminating the blogs and news from each other. The model performances of the two fitted SVMs are provided in Table 6. Although the difference in discriminating blogs from news was not large between the two models, the differences in the f1 scores were still statistically significant: blogs: *t*_(−14.938)_ = 1984.8, *p* < 0.0001, news: *t*_(1959.1)_ = −18.89, *p* < 0.0001, and grand average: *t*_(1, 974)_ = −16.92, *p* < 0.0001. Thus, the results indicated that the SVMs trained on the weighted word frequency provided a better discrimination between the two text varieties. This is the first piece of evidence to support that the weighted word frequency SVMs could derive a more useful set of keywords than the absolute word frequency.

**Table 6:** A comparison of the classification performance of the fitted SVMs.

	**Blogs**		**News**		**Grand average**	
**SVMs**	***M***	***SD***	***M***	***SD***	***M***	***SD***
**F1-score**						
Absolute frequency	0.94	0.01	0.94	0.01	0.94	0.01
Tf-idf weighted frequency	0.95	0.01	0.95	0.01	0.95	0.01

However, the effect of the data representation to the model performance is only one aspect of discriminability. Another aspect is the stability of the representation and the estimated keywords. To this end, we turned to selection frequency, that is, the number of times a given weight and its corresponding word were included among the top 1,000 positive and negative weights estimated during the resampling procedure. An increase in selection frequency indicated that a given keyword was included more often as part of these top weights and, thus, more stable toward small changes in the data.

Interestingly, the SVMs trained on the absolute frequency yielded more top-ranking weights (*w* = 5,030) when compared with the weighted word frequency SVMs (*w* = 4,524). This discrepancy alone indicated that the weighted word frequency SVMs were able to estimate more useful keywords–they remained more stable toward changes in the data and, thus, were more useful indicators of the robust characteristics of text variety. In terms of stability, the average selection frequency was 397.61 (*SD* = 337.88, range: 1–1,000) for the absolute frequency SVMs and 442.09 (*SD* = 391.5, range: 1–1,000) for the weighted word frequency SVMs. The difference was also statistically significant: *t*_(8985.9)_ = 5.9125, *p* < 0.0001. The results indicated that not only did the weighted word frequency SVMs estimate fewer weights, but these same weights were also more stable.

Importantly, the difference in stability between absolute and weighted word frequency was not limited to the whole sets of keywords but was also statistically significant when the keyword sets were limited to the top 100 weights. This is relevant because keyword analysis is typically limited to the top 100 keywords. When considering this part of the distribution, the average selection frequency for the weighted word frequency SVMs was 999.1 (*SD* = 4.54) and 920.6 (*SD* = 15,125) for the absolute frequency ones. This difference was also statistically significant: *t*_(99.18)_ = −5.19, *p* < 0.0001. Thus, the evaluation of the usefulness of the keywords extracted with SVMs has demonstrated that the weighted word frequency provided not only a better discriminability, but also a higher stability of the estimated weights, consequently yielding more stable keywords.

**Figure 2:**
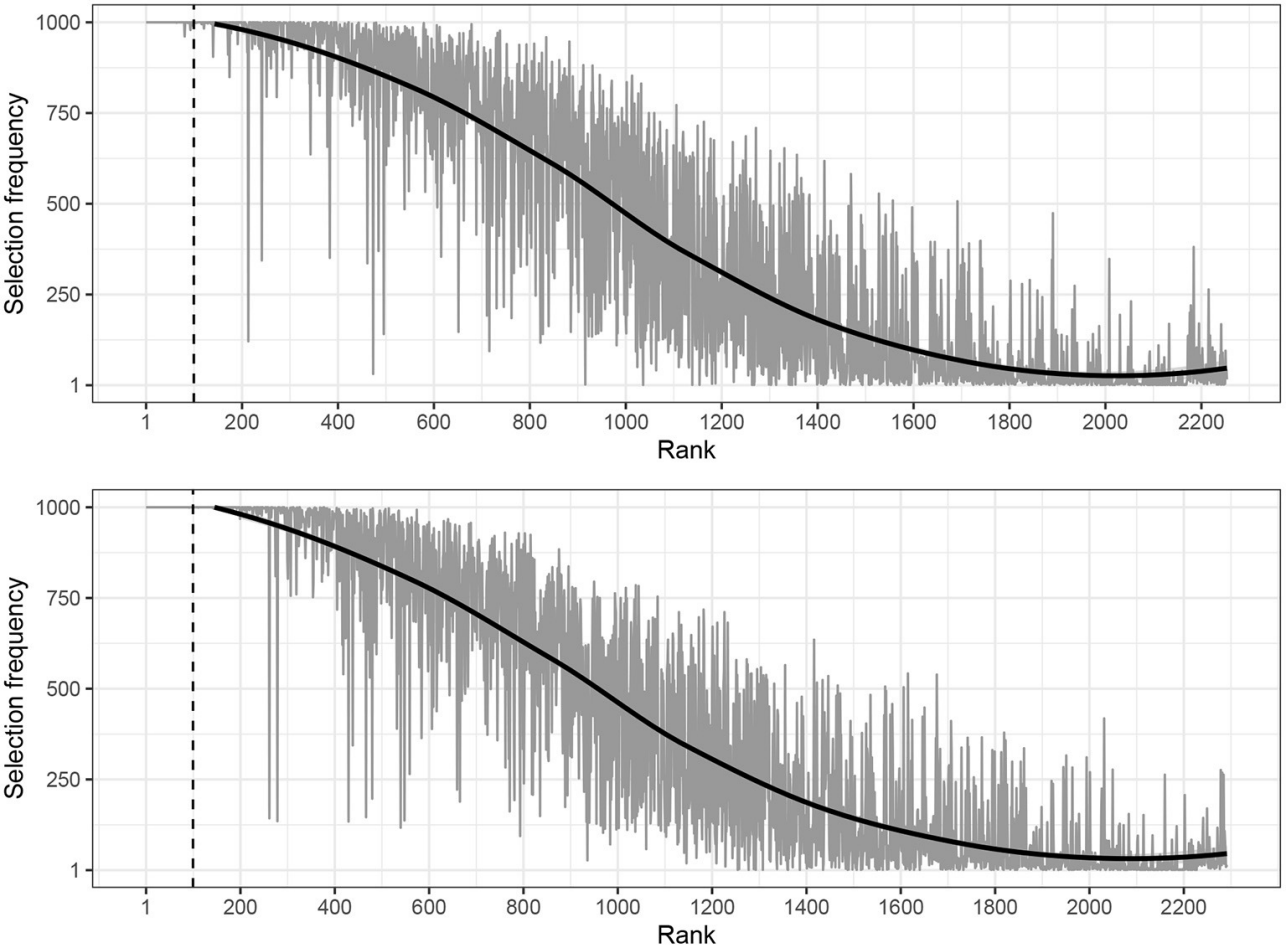
Visualization of the relationship between the selection frequency and rank of the estimated weights presented separately for news **(top)** and blogs **(bottom)** estimated with SVMs (tf-idf). The dashed vertical line indicates the delimiter of the top 100 keyword. The solid black trend line was estimated with loess.

The distribution of the selection frequencies of all the keywords produced with the tf-idf model is provided in Figure 2, in which the estimated weights are on the x-axis in rank order and the selection frequency on the y-axis. For these data, the average selection frequency was 445.96 (*SD* = 445.96) for news and 438.28 for blogs (*SD* = 438.28). As expected, the difference between the text varieties was not statistically significant. For both text classes, we can see that the top 100 keywords had almost perfect selection frequency, indicating that they were very stable across changes in the data. After the 100 top ranking keywords, the stability started to decrease. This also motivated the use of the top 100 keywords for the subsequent analyzes.

In short, we have offered evidence that not only did the weighted BOW representation provide a small but significantly better performance in discriminating between the two text varieties, but it was also accompanied by a substantially better stability of the estimated weights and, thus, the keywords. These results are important in providing a quantitative evaluation of the usefulness of the keywords. Additionally, given that the weighted word frequency representation was evaluated as being more useful, we will only report results using this formatting in the subsequent analyses. In the following section, we move to further validate the estimated weights as proper estimates of keyness.

**Figure 3:**
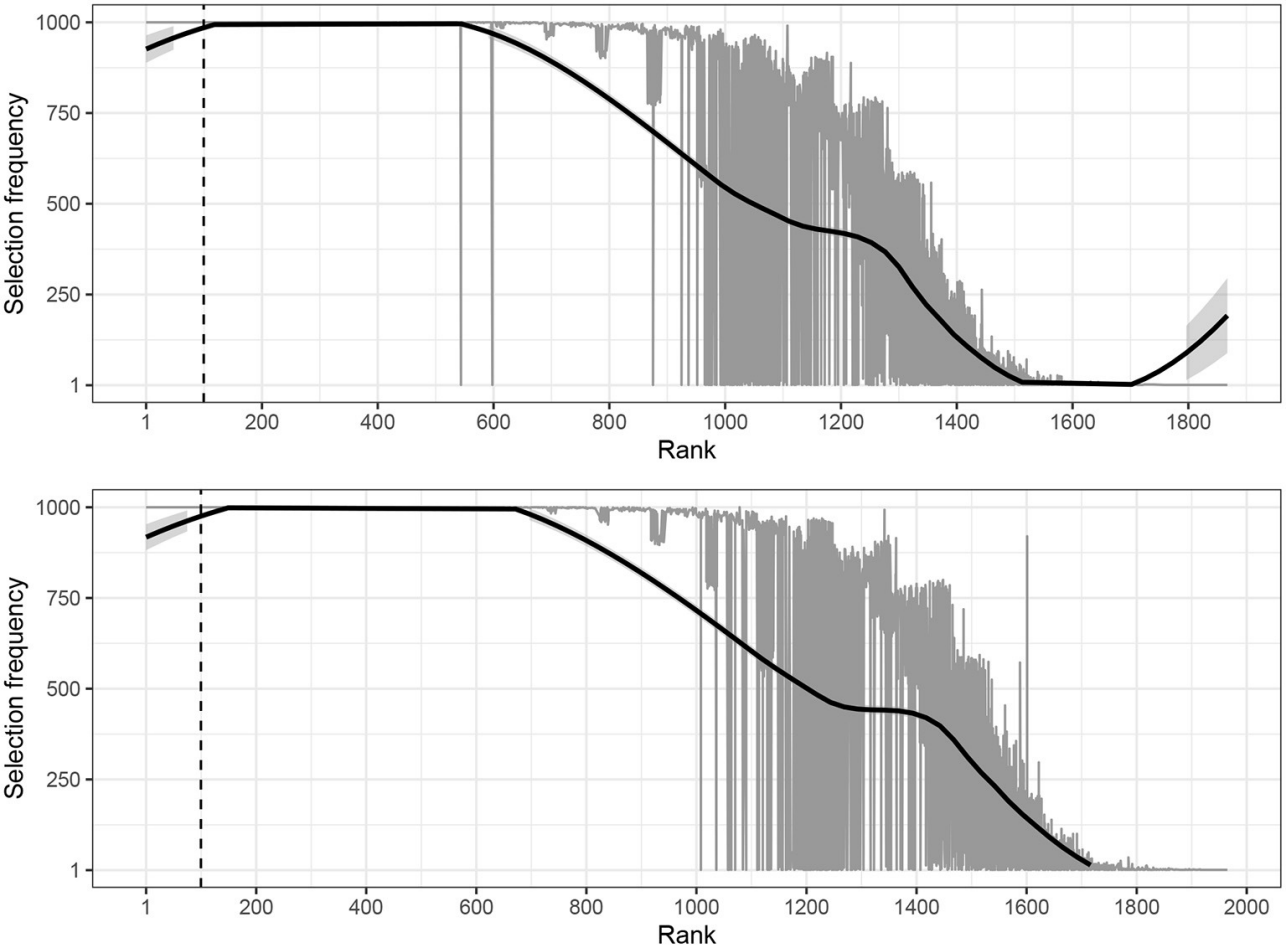
Visualization of the relationship between the selection frequency and rank of the estimated weights presented separately for news **(top)** and blogs **(bottom)** estimated with the TDK. The dashed vertical line indicates the delimiter of the top 100 keyword. The solid black trend line was estimated with loess.

To the best of our knowledge, the stability of the extracted keywords has not been evaluated with traditional keyword methods. The results clearly demonstrated that SVMs produced a smooth functional form between selection frequency and rank as expected for a high-performance discriminative algorithm. In principle, a sampling procedure could be implemented with a traditional keyword method with the caveat that there is no obvious way of determining whether a given sampling size was either too excessive or too lenient. We repeated the process of extracting the keywords with the TDK based on a random sample covering 80% of the original data repeated 1,000 times. The results are visualized in Figure 3.

These results can be considered tentative because the extracted keywords were not optimized for the sample size. Nonetheless, the functional form between selection frequency and rank displayed a fundamentally different shape with the TDK compared to the SVMs (tf-idf). Specifically, the tails of the distribution displayed stronger affinity toward instability with the TDK. However, the differences between the average selection frequencies for news (*M* = 401.12, *SD* = 406.88) and blogs (*M* = 428.61, *SD* = 411.49) compared to the SVMs (tf-idf) were not statistically significant.

The final question pertaining to discriminability is concerned with our choice of model architecture and its potential impact on discriminability, i.e., what was the potential loss in discriminative power when using simpler, linear SVMs compared to more complex models, specifically random forests and BERT. The choice of the encoding schema did not affect the performance of random forests: an average f1-score of 0.92 (*SD* = 0.01) with absolute frequency and an average f1-score of 0.92 (*SD* = 0.01) with tf-idf weighted frequency. This is to be expected as continuous variables are modeled based on ranks and not on the observed values. For these data, random forests had a lower f1-score (grand average) than SVMs with tf-idf weights and the difference was statistically significant: *t*_(1875.3)_ = −59.57, *p* < 0.0001. As expected, BERT provided a better discriminability (*M* = 0.97, *SD* = 0.01) than the linear SVMs. The difference was also statistically significant: *t*_(9.3255)_ = −17.59, *p* < 0.0001. In short, these results indicate that the use of a linear SVMs offer a high performance with a simple architecture for extract keywords without requiring any post-processing of the data.

### 7.2. Usefulness: Distinctiveness and keyness

In this section, we examine the usefulness of the keywords in terms of distinctiveness, that is, the degree to which the keywords reflect the language use associated with their corresponding text variety represented in the target corpus. If the keywords are distinctive, it is expected that their distributional properties mirror the language use associated with their particular text variety. As a first step, we examined the overlap between the keywords for the two text varieties. This can be considered to be a prerequisite to consider the estimated weights as an index of keyness. An overlap between the keyword lists would be indicative of weak distinctiveness because the method would have difficulties in reflecting the language use of the corresponding text variety. The SVMs were able to estimate fully distinctive keywords for the two text varieties because none of the keywords were shared between them. At the same time, it is worth pointing out that this distinctiveness also held with the keywords estimated with the TDK. In short, this demonstrates that the direction of the weights indexed the two text varieties and that the estimated weights can be seen as a good candidate for keyword analysis in terms of their usefulness.

In traditional keyword analysis, the quality of keyness itself is important because it is used to order the keywords; that is, the ranking of the keywords should also reflect the language use represented by their corresponding text variety (Gabrielatos and Marchi, 2011). Thus, the rank order of a given keyword list based on the keyness is expected to be correlated with its corresponding text variety. From this perspective, keywords can be considered distinctive if they are separated from each other by their keyness.

To deepen our understanding of the properties of the estimated weights as a measure of keyness, we further analyzed the difference in dispersion between the estimated weights of SVMs and the LLR scores estimated with the TDK. Furthermore, we focused on dispersion as the TDK was specifically designed to be sensitive to it (for analyzes see Egbert and Biber, 2019). For every keyword estimated with either SVMs or the TDK, we calculated the corresponding text dispersion. Dispersion quantifies the number of occurrences of a given keyword across the texts in a specific text variety (news = *M* = 58.78, *SD* = 95.63; blogs: *M*: 81.88, *SD* = 140.48). There were 1,000 texts per text variety, yielding the theoretical maximum dispersion of 1,000. However, there is a complicating factor with this analysis because these scores are on vastly different scales. For this reason, we used a linear normalization where the keyness scores were normalized to a range between 0 and 1 before the analysis. Additionally, in the case of SVMs, the absolute value of the estimated weights was used in the normalization because their direction only indicated the estimated text variety as either blogs or news.

**Figure 4:**
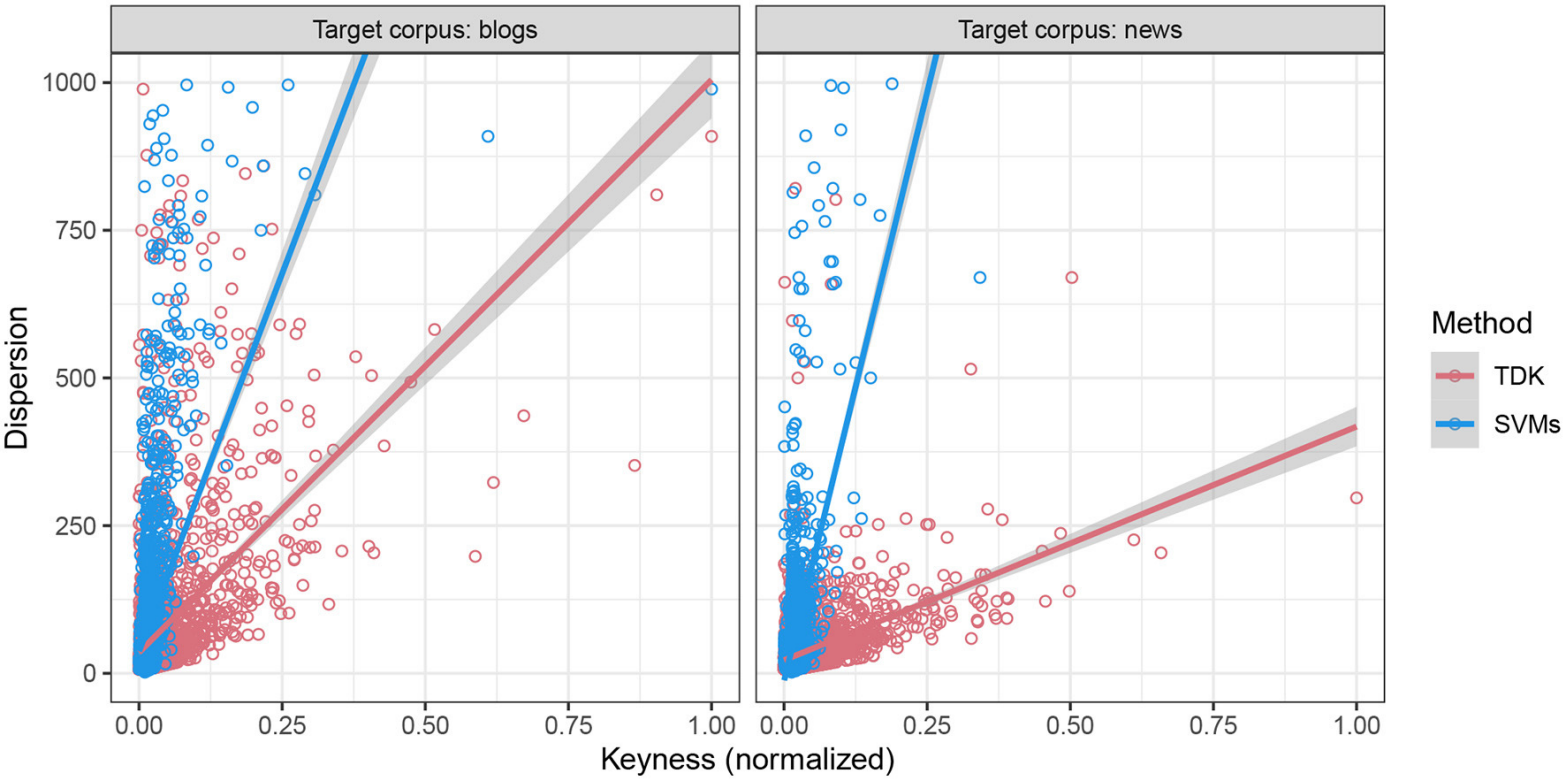
The relationship between dispersion and normalized keyness estimated with SVMs and TDK with 95% CIs. The columns correspond to the target corpus.

In the analysis, we focused on comparing the differences between the methods, here based on the target corpus, because this is the critical part for the analysis when comparing different methods. The data are visualized in Figure 4.

To formally test the difference in the relationship between these two keyness measures and dispersion, we fitted a linear regression model to the data where dispersion was modeled as a function of a three-way interaction: (normalized) keyness, text variety (blogs or news), and method (SVMs or the TDK). This allowed us to avoid carrying out separate subset analyzes of the data as this is known to decrease power and inflate error. The results of the linear regression model demonstrated that the three-way interaction was fully supported by the data based on ANOVA [*F*_(1, 8556)_ = 155.04, *p* < 0.001], offering evidence that the two keyness scores diverged in terms of their distinctiveness. To gain a better understanding of this divergence, we carried out a *post-hoc* linear trend analysis with *p*-values adjusted for multiple comparisons using the Tukey method (Tukey, 1994), as implemented in the R package emmeans, version 1.6.3 (Searle et al., 1980).

The trend analysis examined the strength of the relationship between the keyness and dispersion when the corpus and text variety coincided. This type of an analysis is important in showing the degree to which the keyness score reflects the language use of their respective text variety. In the case of blogs (left panel), the SVMs displayed stronger sensitivity to dispersion (estimate = 2,558, *SE* = 65.4) than the LLR scores (estimate = 969, *SE* = 29.6), and their difference (LLR score − estimated weight) was also statistically significant [estimate = −1, 589, *t*_(8, 556)_ = −22.11, *p* < 0.001]. A similar pattern also emerged in the case of news (right panel), where the trend for SVMs was 3,986 (*SE* = 140.3) and 395 (*SE* = 31.8) for the TDK. Importantly, their difference (LLR score − estimated weight) was also statistically significant [estimate = −3, 591, *t*_(8, 556)_ = −24.96, *p* < 0.001]. This interaction was statistically significant even after removing outliers from the data, i.e., data points which had an absolute residual value >2.5 standard deviations, based on ANOVA [*F*_(1, 8271)_ = 439.34, *p* < 0.001].

In sum, we have offered evidence in this section that the keywords estimated with SVMs are highly distinctive. First, SVMs can produce a list of keywords that are distinctive between themselves, similar to those lists produced by traditional keyword analysis, that is, the TDK. Second, the estimated weights of the SVMs can serve as a measure of keyness, and the score itself reflects the distributional properties of the corresponding text variety. Interestingly, the estimated weights were strongly correlated with dispersion. Together, these properties of the estimated weights are indicative of distinctiveness. In this way, we have demonstrated that the estimated weights are useful not only in discriminating between the text varieties (see Section 7.1), but also in describing the characteristics of the language use associated with the text varieties. Thus, they are capable of distilling even more distinctive aspects of language use when compared to the TDK.

### 7.3. Usefulness: Generalizability to new texts

Generalizability is one of the central questions pertaining to keyword analysis. Are the keywords suitable for describing not only the characteristics of the target corpus used to estimate them in the first place, but also for the characteristics of new texts of the same discourse domain? This was evaluated on both the model performance, as well as on the keywords themselves, here in the two steps outlined below.

First, we turned to evaluating the classification performance of the SVMs in predicting the text variety of new documents. Because we are using machine learning, the fitted SVMs can be used to predict the text variety of a new document. In contrast, traditional keyword analyzes cannot be evaluated based on classification performance as each document has equal status. The TDK was specifically designed to factor in the potential contribution of documents. The use of SVMs allows us to take one step further and evaluate classification performance. This is an important metric to consider if different machine learning algorithms are used to estimate keyness. Although the SVMs achieved a high classification performance, demonstrating that the learned mapping strongly discriminated between blogs and news, it did not necessarily translate to new texts. To test this, we sampled a total of 200 new texts from CORE (secondary corpus). These documents were not used previously either in training or testing with the SVM. The texts were equally split between news (*n* = 100) and blogs (*n* = 100). After preprocessing them using the pipeline described in Section 5.2, the SVMs were used to predict the text variety of a given document. The model performance is summarized in Table 7.

**Table 7:** Model performance of the SVMs on the new texts (*N* = 200) extracted from CORE.

	**Precision**	**Recall**	**F1-score**
Blogs	0.97	0.87	0.92
News	0.88	0.97	0.92

As expected, the classification performance of the SVMs was slightly lower than what is reported in Table 6, but the results indicated that the SVMs provided an excellent fit to the new texts, indicating that the model simply did not overfit the primary data. This offered further evidence that the mapping learned by the SVMs was useful for discriminating between the characteristics of the text varieties.

Second, we moved to examine the extracted keywords. Specifically, we focused on lexical coverage, that is, the extent to which the keywords were used in new texts. Importantly, this index is also suitable for evaluating traditional methods of keyword analysis.

The presence of the keywords is illustrated in Table 8 for SVMs (upper) and TDK (lower). The text is a piece of news reporting on the poverty gap in England. As a typical news article, the text includes frequent reporting verbs often in past tense, such as *said*, perfect aspect, such as *has revealed*, and prepositional phrases, such as *in the North* (see Biber and Egbert, 2018; Biber and Conrad, 2019 for more).

**Table 8:** An excerpt of an article with the top 100 keywords highlighted for news in green and for blogs in red extracted with SVMs (upper part) and TDK (lower part).

Divided nation: Poverty gap in England one of the worst in the Western world
People can now find out how wealthy their area is because a charity has ranked every parish and put the results online
Condemned: Kids born in Toxteth die younger than those in affluent areas
Getty
THE alarming gap between rich and poor neighborhoods makes England one of the most unequal countries in the developed world, research by a poverty charity has revealed. And people are now one click away from finding out how wealthy their area is because the worried charity has ranked every Church of England parish and put the results online. The Church Urban Fund findings show the 10 poorest communities are all in the North. Nine are in the North West with five in - Liverpool. Toxteth (East) where 62% of kids live in poverty is the most deprived parish. Only two of the richest communities are in the North - wags' paradise Alderley Edge in Cheshire, and Wheldrake in York. Camberley Heatherside in Surrey - where only 6% of children and 3% of pensioners live in poverty - is ranked as the richest parish. Paul Hackwood, of the Cuf, said: “we live in one of the most unequal countries in the Western world, where babies born a few miles apart can have widely differing life expectancies - of 10 years or more.” The table of 12,706 Church of England parishes was drawn up using statistics that show life expectancy and poverty.
Divided nation: Poverty gap in England one of the worst in the Western world
People can now find out how wealthy their area is because a charity has ranked every parish and put the results online
Condemned: Kids born in Toxteth die younger than those in affluent areas
Getty
THE alarming gap between rich and poor neighborhoods makes England one of the most unequal countries in the developed world, research by a poverty charity has revealed. And people are now one click away from finding out how wealthy their area is because the worried charity has ranked every Church of England parish and put the results online. The Church Urban Fund findings show the 10 poorest communities are all in the North. Nine are in the North West with five in - Liverpool. Toxteth (East) where 62% of kids live in poverty is the most deprived parish. Only two of the richest communities are in the North - wags' paradise Alderley Edge in Cheshire, and Wheldrake in York. Camberley Heatherside in Surrey - where only 6% of children and 3% of pensioners live in poverty - is ranked as the richest parish. Paul Hackwood, of the Cuf, said: “we live in one of the most unequal countries in the Western world, where babies born a few miles apart can have widely differing life expectancies - of 10 years or more.” The table of 12,706 Church of England parishes was drawn up using statistics that show life expectancy and poverty.

To numerically evaluate the lexical coverage of the keywords in the unseen texts, we calculated it as a proportion where the number of keywords attested to in a given text was divided by the total number of words of that text. Furthermore, as part of the calculation, we only included, those texts that were correctly predicted (92% of the data) because the misclassified texts cannot be used to evaluate the quality of the keywords because we know with certainty that the learned mapping of the model was not sufficient to discriminate between the text varieties associated with these texts. Although the TDK does not provide information about discrimination between the text varieties, the same set of texts were used to keep the setting of the comparison the same. The distributional results based on lexical coverage are given in Table 9 and are broken down by text variety and the number of keywords (all vs. top 100).

**Table 9:** Lexical coverage of the keywords extracted with SVMs and TDK in the unseen texts.

**Lexical coverage**								
	**SVMs**				**TDK**			
	**All keywords**		**Top 100 keywords**		**All keywords**		**Top 100 keywords**	
	***M***	***SD***	***M***	***SD***	***M***	***SD***	***M***	***SD***
Blogs	0.27	0.09	0.08	0.04	0.23	0.07	0.05	0.02
News	0.22	0.06	0.05	0.02	0.12	0.05	0.02	0.01

We focused on the full set of keywords and evaluated the differences between the two methods using a linear regression, in which the lexical coverage was modeled as a function of an interaction between the method (SVMs and TDK) and the text variety (blogs and news). The interaction was statistically significant [*F*_(1, 362)_ = 16.19, *p* < 0.001] with the full set of keywords but not with the top 100 keywords [*F*_(1, 362)_ = 0.32, *p* = 0.57]. In the latter case, only some of the contrasts were statistically significant, which we point out when they are discussed below. Importantly, a *post-hoc* comparison of the contrasts based on the full set of the keywords demonstrated that after adjusting for multiple comparisons, the differences in the average lexical coverage across the text varieties were statistically significant between the two methods (results not shown). Thus, in general, SVMs estimated those keywords that had a higher lexical coverage than the TDK on the news texts on average. Interestingly, the TDK displayed a drastic drop in lexical coverage between all the keywords and the top 100 keywords for news, here with an estimated difference of −0.10 [*t*_(362)_ = −10.43, *p* < 0.001]. This difference was also statistically significant with the top 100 keywords [*estimate* = −0.03, *t*_(362)_ = −8.08, *p* < 0.001]. Thus, regardless of the cut-off point imposed on extracting the keywords, SVMs provided a substantially more robust generalizability for the news texts. For example, only 12% of all the TDK keywords or 2% of the top 100 keywords were attested in the news text variety. This is a low lexical coverage, especially compared with the average number of word types attested in these texts (*M* = 803.74, *SD* = 1523.61).

In short, we have offered evidence that the generalizability of the keywords estimated with SVMs were not limited to the characterization of the texts used in the training, but they extended also to new texts that were not part of the original corpus used for training and evaluating. Additionally, we introduced a measure, which is referred to as lexical coverage, to evaluate the generalizability of the estimated keywords in news texts. This measure is easy to calculate and shows promise because it was able to differentiate between different sets of keywords and methods.

### 7.4. Relevance and keywords

In the previous sections, we have demonstrated that the keywords extracted with SVMs are useful—they allow us to discriminate between news and blogs and refer to the stable and generalizable characteristics of these text varieties. However, the usefulness of these keywords does not necessarily mean that the keywords would be highly relevant for describing the news and blogs texts as instances of their respective text variety. Therefore, in this section, we focus on examining the relevance of the extracted keywords with SVMs and compared these to the TDK. Additionally, to further analyze to what extent the keywords reflected aboutness and other text characteristics, we compared the lexical classes of the extracted keywords. This analysis gave also more information about the lower degree of generalizability of the TDK keywords discussed in the previous section. Similar to previous studies on keyword analysis, we focused on the 100 top keywords associated with each text variety because this analysis relies on the qualitative (dis)similarities between SVMs and the TDK (see Pojanapunya and Todd, 2018; Egbert and Biber, 2019 and citations therein).

#### 7.4.1. Keywords and the characteristics of blogs and news

Previous studies characterizing personal blogs have described them as personal narratives with frequent expressions pertaining to involved and interactive discourse elements and moderate past orientation (Titak and Roberson, 2013; Biber and Egbert, 2016).

Interestingly, when comparing the keywords extracted with SVMs and those identified with the TDK (see Tables 3, 4), the lexical overlap between the two methods was 55%–which was relatively high–indicating that both methods extracted, at least partially, the same set of keywords. Both methods brought to focus the involved oral and narrative aspects associated with the blogs. In particular, this was exemplified by the top ranking of the first person pronouns in the keyword lists: (*i, my, me*) with SVMs and *my, me* with the TDK. At the same time, it is worth pointing out that neither of the methods were capable of fully recovering the complete paradigm of the first person singular pronoun in English among the top 100 keywords: 1) with SVMs, the keyword *mine* had a rank of 215, and 2) with TDK, the keyword *i* had a rank of 1,668. For a method to extract all the relevant keywords, one would expect the complete extraction of a particular category. The keywords extracted with SVMs also covered relatively well the thematic groupings reported by Biber and Egbert (2018) for blogs: Stance, Time/measurement, Description, Personal pronouns, Blogging and Other. Specifically, *love, really, lovely* and *great* could be included in Stance, *day, little* in Time/measurement, *things* in Description, *i, my, me* in Personal pronouns, *blog* in Blogging, and *am, did* in Other. Also the TDK keywords followed these groupings very well, which is logical because the groupings were made from keywords extracted with the same TDK method, though with slightly different settings (see Section 6).

A notable difference between the TDK and SVMs was the larger presence of function words among the keywords extracted with the latter. For blogs, in fact, almost all of the highest-ranking keywords extracted with SVMs were functional, the list including the first person pronouns *i, my, me*, other pronouns *you, we, our, your, it, this*, and past tense auxiliaries or copulas *was, did*. The top keywords extracted with the TDK, in contrast, included first person pronouns and the first person *am*, but also Stance-related words such as *love, lovely, feeling, fun* and Blogging-related words such as *blog, write, things*. Among the keywords extracted with SVMs, these ranked lower because the top positions were occupied by the function words.

To further investigate the differences between the two top 100 keyword lists, we compared the lexical classes associated with them. See Section 2 for more on estimating the lexical classes. The results are presented in Table 10.

**Table 10:** Distribution of the lexical classes among the top 100 keywords.

	**Noun**	**Adjective**	**Function word**	**Verb**	**Other**
**Lexical class for blogs**					
**Method**					
TDK	30	18	12	25	15
SVMs	30	10	30	15	15
**Lexical class for news**					
**Method**					
TDK	55	15	3	10	17
SVMs	48	8	21	10	13

The differences in the distribution of lexical classes between the methods were statistically significant [$X_{\left(4,N=200\right)}^{2}=12.5,p=0.014$]. A residual analysis of the cells indicated that the top 100 keywords extracted with SVMs were driven by a positive association with the function words. Thus, the results indicated that although the lexical overlap between the two keyword lists was high, the differences between them were driven by the higher contribution of function words among the top 100 keywords with the SVMs. This can also explain the lower coverage and smaller generalizability reported for the TDK keywords in the previous section. As opposed to function words, content words that are typical of TDK keywords tend to reflect topical elements of the texts, which are less likely to be shared between different samples, even of the same text variety. We will return to this finding in the general discussion.

News texts are a very typical text variety included in a wide range of language resources. Previous comparative studies on their linguistic characteristics have associated news with the areas of reported communication, information focused, and written discourse (Titak and Roberson, 2013; Biber and Egbert, 2016). These are reflected by very frequent nominal elements, such as nouns, prenominal nouns and modifiers, communication verbs, *that* clauses, and past tense. The text dispersion analysis by Biber and Egbert (2018) identified nine classes for the news keywords: People, Government, Reporting, Figures/ details, Politics, Places, News, and Other.

The top 100 keywords for news with SVMs are given in Table 2 and in Table 5 for TDK. Similar to blogs, the top keywords extracted with SVMs for news included very frequent function words: the pronouns *he, his, its, who, their, they*, the determiners or prepositions *the, in, by, of, an*, and the auxiliary *has* These words do fit the previous analysis on news because the personal pronouns have been associated with narrative, reporting discourse, much like the auxiliary *has* that can co-occur with past tense verbs. The determiners and prepositions refer to nominal and prepositional constructions that have been associated with an information-focused discourse (see also Biber and Conrad, 2019: 218). Of the keywords extracted with SVMs reflecting aboutness, the top ones include the Reporting verbs *said, says, told*, People- and Government-related words *people, government, mr, police, public, obama, president*, and Figure words *million*. Thus, the keywords extracted with SVMs were clearly relevant for news as a text variety, although some, such as the determiner *the*, are also very general.

When compared with the SVM-based keywords, the keywords extracted with the TDK provided a somewhat different set of keywords, the lexical overlap between the two lists was only 39%. Similar to blogs, the TDK keywords included more topical words than the keywords extracted with SVMs. This increase in topicality of the extracted keywords with the TDK was expected, as demonstrated in Section 7.3; they had a lower generalizability in news compared with the keywords extracted with SVMs. There were only three function words among the top 100 TDK keywords, such as *according*, while the others were mostly nominal, such as *government, president, minister* and reporting verbs, such as *said, announced*. To test the positive association of the two keywords lists and function words for news, we compared the distributions of the lexical classes associated with the keywords; see Section 2 for a discussion about the lexical classes. The data are given in Table 10.

The differences in the distributions between the two methods were statistically different [$X_{\left(4,N=200\right)}^{2}=16.64,p=0.002$]. We carried out a residual analysis of the cells, and the results indicated that the difference in the distribution was primarily driven by a positive association between function words and SVMs and a negative association between adjectives and SVMs, respectively. Similar to blogs, we can conclude that the keywords extracted with SVMs had a stronger tendency of containing function words than the TDK. However, this difference in the distribution of the content words was related to adjectives, at least in these data.

In sum, we have demonstrated that SVMs are capable of extracting keywords that are relevant for their corresponding text variety. Additionally, they also overlap lexically to a greater extent than those keywords extracted with the TDK. At the same time, the analysis presented here has also shown that SVMs tended to extract keywords including a relatively larger proportion of function words among the top 100 keywords than the TDK, especially in the case of news. We will return to this point in the general discussion.

## 8. General discussion

In the current study, we have approached keyword analysis from the perspective of predictive modeling. Specifically, we introduced linear SVMs as a method for exploring keyness and demonstrated their utility as part of text analysis in corpus linguistics in general. They offer interpretable and linguistically motivated results with strong discriminative performance. We have demonstrated how predictive modeling can be used to extract keywords, that is, predictive keywords. This approach has two clear benefits. The first benefit is related to the process of the predictive model itself; namely, it allows us to evaluate the degree to which the texts associated with the target corpus are discriminated from the reference corpus. This is important because it provides us with information about the typicality of the texts as exemplars of their corresponding discourse domain relative to the reference corpus. A traditional keyword analysis assumes that all the texts are equal in their typicality, i.e., a given document is more or less representative of its corresponding category. While the TDK is based on dispersion and, thus, sensitive to distributional properties associated with individual documents, only predictive modeling allows us to take steps toward evaluating the representativeness of individual documents as members of their category—a direction facilitating a more rigorous quantitative text analysis.

The second benefit pertains to keyness associated with a particular discourse domain and to its evaluation. There are a number of challenges related to keyword analysis and how to evaluate the quality of extracted keywords. Although the utilization of keyword analysis has a long tradition in corpus linguistics, a systematic approach for evaluating them is, nonetheless, absent from most prior studies. For more on this, see the introduction. To tackle this issue, we approached the evaluation from the point of view of variable selection in machine learning, specifically anchoring it relative to the concepts of usefulness and relevance. In this approach, usefulness can be understood as referring to the set of variables that retain high predictive power. In contrast, relevant variables can be understood as related to the set of variables that provide descriptive adequacy of the categories under investigation. Adopting this approach provided us a direct way to contrast the keywords extracted with different methods. To compare the quality of the keywords extracted with SVMs, we used the method proposed by Egbert and Biber (2019) as a point of comparison because it has been demonstrated to extract keywords of high quality. Finally, the approach to evaluate keywords is not only informative about their quality, but it can also be used to reveal how the keywords extracted with various methods may differ.

In the current study, we demonstrated that although the keywords extracted with SVMs and the TDK were partially identical (see Section 7.4), there were significant differences between them. This indicates that these two methods focused on different aspects of the discourse domain, as attested to in the target corpus and the reference corpus. In general, SVMs were found to have higher degree of usefulness than the TDK. Usefulness was specifically anchored in relation to four concepts: 1) discriminability, 2) stability, 3) distinctiveness, and 4) generalizability. Because SVMs learn a mapping to discriminate between texts in the target corpus and reference corpus, the properties of the keywords extracted with SVMs also reflect this process and tend to display those qualities that maximize usefulness. In this study, we have proposed general concepts for evaluating usefulness. In future studies, additional tests can be easily incorporated such as evaluating discriminability in terms of word frequency or lexical coverage in terms of word type frequency.

Out of the four concepts related to usefulness, stability is a property that is effectively absent from previous studies. The analysis based on stability brought forward an interesting finding. Specifically, the top 100 keywords extracted with SVMs effectively remained the same, regardless of the small changes in the distribution of the data. This is a desirable quality because it ensures that the keywords are likely to be applicable to new texts associated with a given discourse domain. Importantly, the stability of the keywords is correlated with the rank of the keyword, and the instability steadily increases with the rank.

In terms of relevance, the analysis showed that both SVMs and the TDK extracted keywords that were linguistically motivated, reflecting the previously reported characteristics of texts associated with blogs and news. However, the keywords extracted with the TDK tend to contain a higher number of content words than SVMs. This property can also be used to motivate the discrepancy of these two methods in generalizability because we showed that SVMs retained better generalizability to new texts in the same discourse domain when compared with the TDK. Previous studies have shown that topical elements tend to lack generalizability (see Laippala et al., 2021, and citations therein). In contrast, the proportion of function words among the keys was slightly higher with SVMs. This suggests that the TDK is more likely to bring forth aspects related to the aboutness of the discourse domain associated with the texts, while SVMs also reflect the syntactic and functional characteristics of the discourse domain.

In sum, we have shown that the proposed approach not only allows one to evaluate the quality of the extracted keywords, but it also provides the opportunity to gain a better understanding of a given method and its ability to extract keywords. Although we have demonstrated that the keywords extracted with SVMs tend to have a number of desirable properties, there is one key limitation that should be kept in mind. This limitation is related to using machine learning to extract keywords in general and is not specific to SVMs. A traditional keyword analysis utilizes a reference corpus that tends to be significantly larger in size than the target corpus. In a machine learning setting, a specific model is first chosen and then utilized to discriminate among the texts associated with the target corpus from the reference corpus. A significant imbalance in size between the two corpora is likely to make the modeling of the data difficult. For example, the model may display a poor discriminative power between the two corpora. If the model is unable to separate the texts associated with the two corpora from each other, it is likely that the keywords extracted from the model would lack quality. At the same time, it is not clear whether the keywords extracted with a traditional method such as the TDK would retain a higher quality in this kind of setting. In studies that utilize keyword analysis, a comprehensive analysis of the quality of the extracted keywords is rarely adopted. This, in and of itself, is an interesting question that should be pursued in future keyword analysis research. Another potential direction for future research is concerned with the relationship between aboutness and topicality. This type of analysis would be firmly situated within the concept of relevance.

## Data availability statement

The original contributions presented in the study are included in the article/supplementary material, further inquiries can be directed to the corresponding author.

## Author contributions

A-JK and VL contributed equally to the current study and approved the final version. Both authors contributed to the article and approved the submitted version.
